# Quality of life in patients with neurofibromatosis type 1 and 2 in Canada

**DOI:** 10.1093/noajnl/vdaa003

**Published:** 2020-01-10

**Authors:** Geohana Hamoy-Jimenez, Raymond Kim, Suganth Suppiah, Gelareh Zadeh, Vera Bril, Carolina Barnett

**Affiliations:** 1 Division of Neurology, Department of Medicine, University Health Network and University of Toronto, Toronto, Ontario, Canada; 2 Division of Medical Oncology, Department of Medicine, University Health Network and University of Toronto, Toronto, Ontario, Canada; 3 Division of Neurosurgery, Department of Surgery, University Health Network and University of Toronto, Toronto, Ontario, Canada; 4 Institute of Health Policy, Management and Evaluation, Dalla Lana School of Public Health, University of Toronto, Toronto, Ontario, Canada

**Keywords:** EQ-5D-5L, NF1, NF2, pain interference, quality of life

## Abstract

**Background:**

There is scarce data on the quality of life of people with neurofibromatosis type 1 (NF1) and type 2 (NF2) in Canada.

**Methods:**

A cross-sectional study of adults with NF1 and NF2 attending a tertiary center. Patients completed generic measures (SF-36, EQ-5D-5L, and PROMIS pain interference) and disease-specific questionnaires (PedsQL NF1 module and the NFTI-QOL for NF2). We compared generic scores between NF1 and NF2 individuals and used regression models to assess factors associated with quality of life.

**Results:**

Hundred and eighty-four participants were enrolled. Mean age was 33 years in NF1 and 40 years in NF2. NF1 and NF2 individuals had lower employment rates and lower scores in all domains of the SF-36 compared to the general Canadian population (*P* < .005). Using the EQ-5D-5L, there was a high proportion of pain (64% in NF1 and 74% in NF2) and anxiety/depression (60% in NF1 and 68% in NF2). Pain interference correlated with poor quality of life in NF1 and NF2; perceived physical appearance was the main predictor of mental well-being in NF1.

**Conclusions:**

Individuals with NF1 and NF2 have low quality of life, and this correlates with pain, anxiety, and depression, which are prevalent in NF1 and NF2. Perceived physical appearance predicts quality of life in NF1. A multidisciplinary approach is necessary for patients with NF1 and NF2, including mental health and pain management.

**Importance of the Study**

There is limited data on the quality of life of individuals with NF1 in Canada. In this cohort of young individuals, we found a high number of individuals who were unemployed or on disability. We also found reduced quality of life in NF1 and NF2 patients, and together these findings show the high burden of NF1 and NF2. In both conditions, the main factor affecting the quality of life was pain. We also found that a large number of individuals with NF1 and NF2 reported anxiety or depression. In addition, in NF1, self-perceived physical appearance—rather than visibility rated by a clinician—was the main predictor of mental well-being. Our findings highlight the importance of multidisciplinary care for people with NF1 and NF2, including assessing mental health and pain.

**Key Points**

- People with NF1 and NF2 have worse quality of life than the general Canadian population.
- Pain, anxiety, and depression are common in individuals with NF1 and NF2.
- Mental health and pain care are important aspects of care for people with NF1 and NF2.

Neurofibromatosis type 1 (NF1) and type 2 (NF2) are hereditary, tumor predisposition syndromes that affect multiple systems. NF1, caused by pathogenic variants in the *NF1* gene, is characterized by café au lait macules, skin-fold freckling, Lisch nodules, optic glioma, and bone deformities. In addition, NF1 patients are prone to developing innumerable peripheral nerve sheath tumors. Cutaneous neurofibromas, while benign, have significant psychosocial implications due to itchiness, physical disfigurement, and sometimes pain. Similarly, plexiform neurofibromas often cause pain, deformity, and can also develop into a highly aggressive sarcoma called malignant peripheral nerve sheath tumors (MPNSTs).^1^

Patients with NF2 typically develop bilateral acoustic schwannomas, as well as schwannomas on other nerves, meningiomas, and early cataracts. Clinically, these manifest with symptoms such as progressive hearing and visual loss, facial weakness, balance and mobility impairments, and pain.^2^ These patients harbor pathogenic variants in the *NF2* gene.

Health-related quality of life (QoL) is a complex multidimensional construct that depends on biological and psychological variables, symptoms, functional status, general health perceptions, and the influences of personal (eg, individual preferences) and environmental factors (eg, social support), as well as nonmedical factors.^3^ The clinical manifestations of NF1 and NF2 have profound effects and limitations on daily life activities, social roles, and mental health. As a result, it is not surprising that affected individuals may have poor QoL. Several studies using generic and disease-specific measures have shown reduced QoL among patients with NF1.^4–6^ Disease visibility and whole-tumor burden have been recognized as major predictors of poor QoL in NF1,^5,7^ as well as pain interference in young patients with NF1 and plexiform neurofibromas.^8^ There are fewer studies assessing QoL in individuals with NF2. A qualitative study found 11 domains affecting QoL in patients with NF2: hearing, balance, facial function, vision, oral intake, future uncertainty, psychosocial, cognition, sexual activity, pain, and vocal communication.^9^ Additionally, one study in the United Kingdom and one in the United States reported lower SF-36 scores in NF2 patients compared to the general population.^10,11^ One study assessed 288 patients with NF2 and found worse scores on items reflecting dizziness and balance problems, hearing loss, and impact of NF2 on role and outlook on life.^12^

Given the lack of studies assessing the QoL of patients with NF1 and NF2 in Canada, we conducted a cross-sectional study of QoL in patients attending an academic clinic for adults with neurofibromatoses. We hypothesized that patients with NF1 and NF2 have a low QoL compared to the Canadian general population. We also hypothesized that specific disease manifestations, such as disease visibility and pain in NF1, and hearing loss in NF2, would correlate with overall QoL.

## Methods

This is a cross-sectional study of patients attending the Elisabeth Raab Neurofibromatosis Multidisciplinary Clinic at Toronto General Hospital, between January 2016 and December 2017. We invited all adults who meet the clinical diagnostic criteria^13,14^ and/or genetically confirmed NF1 and NF2 to participate. We excluded from the study individuals unable to complete the questionnaires (eg, language barrier or limited literacy). The University Health Network research ethics board approved the study, and all participants provided written informed consent.

The attending clinician completed a standardized form for clinical manifestations of NF1 or NF2, including a family history of NF, genetic testing, known plexiform neurofibromas (either visible on examination by MRI), presence of spinal and brain tumors, other malignancies, and history of MPNSTs. We did not ask clinicians to specify if the plexiforms were diagnosed by imaging or examination, and it was a yes/no question. For individuals with NF1, the clinicians also rated the disease visibility using the Ablon visibility index.^15^ In this measure, patients are assessed while fully dressed, and visibility ranges from 1—mild, no visible tumors outside normal clothing areas to 3—indicating numerous visible tumors in the face or neck, or visible limp. For NF2, the physicians also completed the House and Brackmann facial nerve scale^16^ that rates facial nerve impairments from 1—normal symmetric function to 6—indicating complete paralysis. Finally, the physicians rated the hearing of NF2 patients, based on the last audiogram, using a 5-item Likert scale ranging from normal hearing to profound hearing loss.^17^

The patients completed several questionnaires, including demographic data (age, sex, employment, and educational status), and health-specific measures described below.

### Generic Measures

*SF-36*: This is a 36-item generic QoL measure^18^ that has normative data for the Canadian population.^19^ The SF-36 has 8 domains: physical functioning, role physical, bodily pain, general health perception, energy/vitality, social functioning, role emotional, and mental health. Additionally, the physical health domains are summarized in a physical score (PCS) and the mental domains in a mental score (MCS). Lower scores indicate lower QoL. The SF-36 has been widely used and validated in the Canadian population, with available normative data.^19^

*EQ-5D-5L*: This is a 5-item multi-attribute questionnaire.^20^ The EQ-5D-5L dimensions are mobility, usual activities, self-care, pain, and depression/anxiety, each with 5 response options. The EQ-5D-5L can be scored as health utilities, where a score of 1 indicates perfect health and 0 indicates death; negative scores are possible, indicating states valued as worse than death. Additionally, the EQ-5D-5L has a visual analogue scale (VAS) for a direct valuation of general health, ranging from 0—worst possible health to 100—best possible health. This has been widely used and validated in Canada and we used a Canadian valuation algorithm to estimate the EQ-5D-5L utility scores.^21^

*PROMIS pain interference short form 8a*: This is an 8-item questionnaire, assessing how pain affects social, cognitive, physical, and recreational activities. Raw scores are converted into T-scores, ranging between 40.7 and 77; higher scores indicate worse pain interference.^22^ This measure has been recommended for trials assessing pain in adults with NF1, although it has not been specifically validated in Canada.^23^

### Disease-Specific Measures

*PedsQL NF1 adult module*: This is a 74-item self-reported measure, specifically developed for NF1.^24^ The PedsQL NF1 module has 16 domains assessing physical, emotional, social, and cognitive functioning, as well as communication, worry, perceived physical appearance, pain and hurt, paresthesia, skin irritation, sensation, movement and balance, daily activities, fatigue, treatment anxiety, and sexual functioning. Each item is scored in a 5-option Likert scale ranging from 0—never to 4—almost always and then reverse-scored between 0 and 100. The domain scores are calculated as the average score for all the items in each domain, and a total score is obtained by calculating the average of all items. Higher scores indicate better QoL. To our knowledge, this measure has not been previously used or validated in Canada.

*NFTI-QoL*: This is an 8-item NF2-specific questionnaire.^25^ The items reflect balance, hearing, facial weakness, visual problems, mobility, role and outlook on life, pain, and anxiety/depression. Each item is scored on a 4-point Likert scale from 0 to 3, and the total score is the sum of all items. Higher scores indicate worse QoL. To our knowledge, there are no previous Canadian data for this measure.

### Statistical Analysis

Continuous data are described as mean ± standard deviation, or median and interquartile range; categorical data are presented as number and proportion.

For the generic measures (SF-36, EQ-5D-5L, PROMIS pain interference), we compared the mean scores between NF1 and NF2 individuals using *t* tests. For the SF-36, we also compared the results to Canadian normative data.

For the PedsQL NF1, we calculated the mean score in each dimension as well as the total score. For the NFTI-QoL, we calculated the mean total score and also the median score for each item.

We tested the correlation coefficients between the different generic and disease-specific measures. Finally, we conducted multivariable regression analyses to study the relationship between the different clinical and demographic variables and QoL. Because we used different QoL measures, we decide a priori to build regression models for the SF-36 PCS, SF-36 MCS, and total PedsQL NF1 score. If enough patients were enrolled with NF2, we also planned to build a model for the NFTI-QoL. We chose the variables for the models based on clinical grounds and previous reports, and we tested all variables for collinearity and possible interactions. We removed variables that were highly collinear, and we retained interaction terms according to likelihood ratio tests. For each dependent variable, we chose the best performing model, comparing *R*^2^ statistics and residuals. Final models were bootstrapped with 100 repetitions to account for overfitting.

All analyses were done with R statistical software version 3.5.1, and we considered *P*-values <.05 as significant.^26^ We used Bonferroni correction when appropriate.

## Results

Between January 2016 and December 2017, we screened 292 individuals with NF1 and 46 with NF2. A total of 184 individuals agreed to participate, 162 (88%) had NF1 and 22 (12%) had NF2. The mean age was 33 ± 13 years in NF1 and 40 ± 15 years in NF2; the proportion of females was similar between groups: 89(55%) in NF1 and 13 (59%) in NF2. Thirty-four (22%) of the individuals with NF1 reported being unemployed or receiving disability benefits and this was reported in 9 (45%) in the NF2 group. Detailed demographic and clinical data are described in Table 1.

**Table 1.** Demographic Profile of NF1 and NF2 Patients

	NF1 (*n* = 162)	NF2 (*n* = 22)
AGE (mean ± SD)	33 ± 13.5	40 ± 15.2
Female/male	92 (57%)/70 (43%)	13 (59%)/9 (41%)
Marital status^a^		
Single	101 (65%)	13 (59%)
Married/common law	47 (30%)	7 (31%)
Divorce/separated	4 (2.5%)	1 (5%)
Other (living with partner, widow)	4 (2.5%)	1 ((5%)
Highest education^b^		
Some or completed primary	5 (3%)	1 (4%)
Some or completed secondary	34 (22%)	2 (9%)
Some or completed trade or community program	57 (37%)	9 (41%)
Some or completed university	58 (38%)	10 (46%)
Work status^c^		
Employed	75 (49%)	10 (50%)
Unemployed	10 (6%)	4 (20%)
Disability	24 (16%)	5 (25%)
Student	35 (23%)	1 (5%)
Other	9 (6%)	0 (0%)
History of MPNST	14 (9%)	NA
Optic glioma	25 (15%)	NA
Known plexiform neurofibroma	63 (39%)	NA
Ablon’s index (median, range)	2 (1–3)	NA
Hearing		
Normal	NA	8 (36%)
Mild to moderate hearing loss	NA	7 (32%)
Severe or profound hearing loss	NA	7 (32%)
Facial nerve scale		
Grade 1	NA	14 (63%)
Grades 2 and 3	NA	5 (23%)
Grades 5 and 6	NA	3 (14%)

MPNST, malignant peripheral nerve sheath tumor.

^a^156 patients with NF1 and 22 with NF2 completed the marital status question.

^b^154 patients with NF1 and 22 with NF2 completed the education question.

^c^153 patients with NF1 and 20 with NF2 completed the employment question.

**Figure 1.**
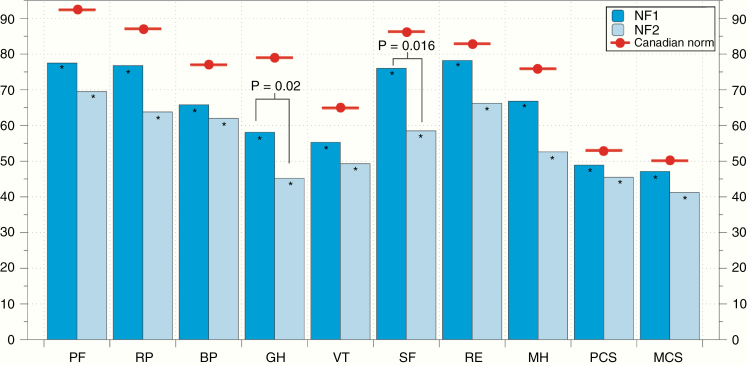
Mean SF-36 scores in patients with NF1 and NF2, compared to the Canadian general population. NF1 and NF2 patients had significantly lower scores in all dimensions of the SF-36 compared to Canadian normative data (red dots, *P* < .005 for all domains). When comparing NF1 and NF2, only global health and social functioning were significantly lower in NF2 than NF1 (*P*-values .02 and .016, respectively).

Patients with NF1 and NF2 had significantly lower scores in all the domains in the SF-36 compared to the Canadian normative population at this age range (*t* test *P* < .005). In general, NF2 patients had lower scores than patients with NF1, although this difference was only significant for Social Functioning and General Health (*P*-values .02 and .016, respectively). The comparison of the SF-36 scores of the NF1 and NF2 participants in relation to Canadian normative data is shown in Figure 1, and the specific SF-36 scores are given in Supplementary Table 1.

**Figure 2.**
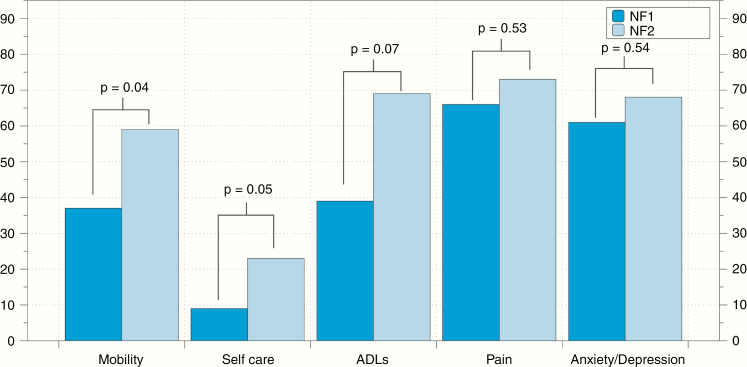
Proportion of EQ-5D-5L domains affected in individuals with NF1 and NF2. More than 60% of NF1 and NF2 patients reported some degree of pain or anxiety/depression. Individuals with NF2 reported significantly higher prevalence of mobility problems and difficulties with self-care activities than those with NF1.

When analyzing the EQ-5D-5L, we found that a high proportion of patients reported some degree of pain/discomfort (66% for NF1 and 73% NF2, respectively) and anxiety/depression (61% and 68% for NF1 and NF2, respectively). The distribution of affected EQ-5D-5L scores is shown in Figure 2. The mean EQ-5D-5L VAS score was 74.5 ± 23 for NF1 patients and 64.2 ± 21 for NF2 (*P*-value .05). The mean EQ-5D-5L health utility score was 0.73 ± 0.24 for NF1 and 0.65 ± 0.25 for NF2 patients (*P*-value .16).

The PedsQL NF1 domains had less than 10% missing values, with the exception of the sexual domain that was missing in 20 (18%) of the patients. We obtained total PedsQL NF1 scores for 153 patients. Cronbach’s alpha for the PedsQL NF1 was 0.96. The PedsQL NF1 domains with the lowest scores (worse QoL) were perceived physical appearance, worry, cognitive, emotion, fatigue, and pain. Supplementary Table 2 shows the mean PedsQL scores by domain.

When looking at the NFTI-QoL, we only had one missing item in one patient, and we could obtain total scores for all patients; Cronbach’s alpha was 0.87. The items with highest (worse) scores were hearing (median 2, range 1–3) and outlook on life (median 2, range 0–3); these items, as well as the item on balance, had the lowest floor effects. The total NFTI-QoL had moderate to high correlations with the EQ-5D-5L utility score (*r*: −0.76, CI: −0.89, −0.49, *P* < .001), the EQ-5D-5L VAS score (*r*: −0.52, CI: −0.77, −0.11, *P* = .01), SF-36 PCS (*r*: −0.65, CI: −0.86, −0.26, *P* = .003), and the SF-36 MCS (*r*: −0.63, CI: −0.85, −0.23, *P* = .004). The distribution of item scores is shown in Supplementary Figure 1.

**Figure 3.**
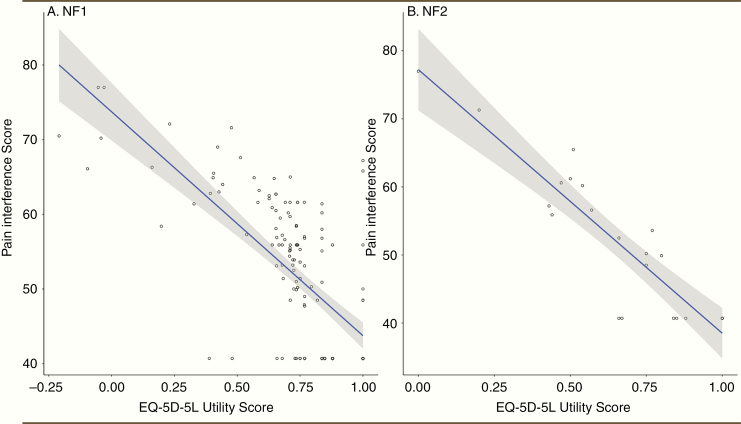
Correlations between pain interference scores and ED-5D-5L utility scores in individuals with (A) NF1 and (B) NF2. There were high correlations between pain interference scores and health utilities for both NF1 and NF2 patients, indicating that pain is a major driver of QoL in individuals with NF.

The PROMIS pain interference scores had moderate to high correlations with the SF-36 PCS, EQ-5D-5L health utilities, global health VAS, and the NFTI-QoL pain item. When analyzed separately by type of NF, the correlation between pain interference and health utilities was −0.70 (CI: −0.77, −0.61, *P* < .001) for NF1 and −0.90 (CI: −0.96, −0.78, *P* < .001) for NF2, as shown in Figure 3. The correlation matrix can be found in Table 2.

**Table 2.** Pearson Correlation Coefficients of the Total PedsQL Scores, Pain Interference, EQ-5D-5L, and SF-36 Scores

	PedsQL-NF1 total score	PROMIS pain interference	EQ-5D-5L utilities	EQ-5D-5L VAS	SF-36 PCS	SF-36 MCS
PedsQL total score	1.00					
PROMIS pain interference	−0.58 (−0.68, −0.47)^a^	1.00				
EQ-5D-5L utilities	0.57 (0.46, 0.68)^a^	−0.70	1.00			
EQ-5D-5L VAS	0.41 (0.26, 0.54)^a^	−0.53 (−0.63, −0.42)^a^	0.47 (0.34, 0.58)^a^	1.00		
SF-36 PCS	0.63 (0.52, 0.72)^a^	−0.68 (−0.76, −0.59)^a^	0.60 (0.49, 0.69)^a^	0.47 (0.34, 0.58)^a^	1.00	
SF-36 MCS	0.49 (0.36, 0.61)^a^	−0.23 (−0.37, −0.08)	0.32 (0.17, 0.45)^a^	0.20 (0.04, 0.35)	0.15 (−0.00, 0.30)	1.00

MCS, mental component score; PCS, physical component score; VAS, visual analogue scale.

Bonferroni corrected *P*-value = .003.

^a^ *P* < .003.

With 162 NF1 patients, we could fit regression models with up to 16 variables, using the common rule of thumb of 10 patients per variable^27^; however, we aimed at a maximum of 10 variables to avoid overfitting and to test interaction and nonlinear terms. Given the small number of NF2 patients, we did not perform regression analysis in this group. The final model for the SF-36 PCS had age, sex, pain interference score, known plexiform neurofibroma, known spinal tumor, optic glioma, history of MPNST, and history of other cancers, with *R*^2^: 0.60 and *P* < .001. For the SF-36 MCS, the final model had age, sex, pain interference score, known plexiform neurofibroma, optic glioma, history of MPNST, other cancers, employment status, marital status, and perceived physical appearance score, with *R*^2^: 0.36 and *P* = .0003. For the PedsQL model, the variables were sex, age, pain interference score, plexiform neurofibroma, optic glioma, MPNST, and other cancers, with *R*^2^: 0.44 and *P* < .0001. Worse pain interference scores and older age were significantly associated with worse SF-36 PCS, while worse perceived physical appearance and being divorced were associated with worse SF-36 MCS. For the PedsQL scores, being female, older, and with higher pain interference scores, was associated with worse QoL. Regression estimates for SF-36 PCS and MCS are given in Table 3, and regression estimates for the PedsQL are given in the Supplementary Material.

**Table 3.** Regression Estimates for the SF-36 Physical and Mental Component Scores in NF1

SF-36 PCS Model				SF-36 MCS Model			
	Estimate	SE	*P*-value		Estimate	SE	*P*-value
Intercept	89.4	5.3	<.0001*	Intercept	25.73	12.7	.04*
Sex	0.37	1.98	.85	Sex	0.09	2.50	.97
Age	−0.15	0.07	.04	Age	0.06	0.09	.54
Pain interference	−0.68	0.09	<.0001*	Pain interference	−0.11	0.12	.39
Known plexiform	1.40	1.98	.48	Known plexiform	2.26	2.29	.33
Optic glioma	−1.33	2.90	.62	Optic glioma	1.19	3.15	.70
MPNST	2.21	−1.98	.57	MPNST	1.98	4.54	.66
Other cancers	−6.40	3.22	.05	Other cancers	−5.35	3.43	.12
Spinal tumor (yes/no)	−1.65	1.98	.41	Perceived physical	0.13	0.04	.0003*
				Employed	−1.57	3.4	.64
				Unemployed	1.35	3.6	.5
				Other employment	1.36	4.8	.77
				Married/partner	17.5	7.8	.028*
				Single	17.1	7.73	.03*

PCS, physical component score; MCS, mental component score.

MPNST, malignant peripheral nerve sheath tumor (yes/no); perceived physical: perceived physical appearance subscore, from PedsQL NF1 module; married/partner and single, compared to being divorced; employed, unemployed, and other employment, compared to being on disability.

*P < .05.

## Discussion

We found that patients with NF1 and NF2 attending a tertiary academic center in Canada have reduced QoL, and this finding was consistent across the different QoL measures used.

This cohort had low employment rates, 49% in NF1 and 50% in NF2. These are lower than the employment rates for the general Canadian population at this age range, which was 83% in 2018.^28^ This provides indirect evidence of the social impact of NF1 and NF2, with only half of the individuals participating in the workforce. This is particularly striking as our cohort was young, although it may also reflect more severe patients seen at a tertiary center.

Additionally, our NF1 population had overall a higher than expected level of educational attainment. A large, population-based study in Denmark showed that 21% of individuals with NF1 completed post-secondary education,^29^ lower than our cohort, where 37% reported some or completed trade or community program and 38% some or completed university program. This could be due to the exclusion of individuals who could not complete the questionnaires, for example, due to low literacy or language barriers, thus resulting in selection bias toward those with higher education levels. Additionally, it is possible that individuals with learning difficulties would choose not to answer a long questionnaire, such as in this study. Given that this cohort was in average well educated, the lower employment rate is unlikely due to learning difficulties and more likely due to physical disabilities, chronic pain, and mental health problems. In our cohort, 16% of patients with NF1 and 25% with NF2 reported being on disability; this is higher than the approximately 5% of residents of the province of Ontario receiving government disability benefits in 2017.^30^ Additionally, individuals with NF1 may face discrimination based on the cutaneous manifestations of the disease, which may also affect employment rates.

In our study, and using the SF-36, both NF1 and NF2 patients had significant lower scores in all the domains, compared to the Canadian general population. This is in keeping with the findings of previous studies in different countries such as France, Italy, and the United States.^4,5,31^While there was a trend toward lower scores in NF2, we only found significant differences between NF1 and NF2 patients in social functioning and general health. This is in contrast to the findings of Merker et al.,^11^ who found that the mental component score was more affected in NF1 patients, while NF2 patients had lower scores in the physical component. However, our NF2 cohort was considerably smaller than the NF1 group, and this could affect the likelihood of finding a statistically significant difference.

Using the EQ-5D-5L, we found a high prevalence of self-reported pain in N1 and NF2 patients. Importantly, pain interference was the main driver of QoL both for NF1 and NF2, demonstrated by the high correlations between pain interference and QoL scales. In the case of NF1, multivariable analyses showed that pain interference was one of the main variables affecting the physical component of the SF-36, as well as total PedsQL scores. Previous studies have shown that pain interference affects QoL in patients with NF1 and plexiform neurofibromas,^8^ but our study shows that pain is widely prevalent and clinically important in patients with NF1, regardless of the presence of plexiform neurofibromas, as shown in our regression models. Due to the numerous questionnaires, and to minimize patient burden, we did not specifically ask about the sources of pain, so we could not stratify by pain cause, and this was beyond the scope of this study. Additionally, we also found that pain is a prevalent problem for individuals with NF2, with high impact on overall QoL. Pain in NF2 has not been as widely reported as in NF1, but there are potential causes including NF2-related neuropathy, which has been described in up to 60% of NF2 individuals.^32^ Additionally, during development of the NFTI-QoL, patient focus groups identified pain as a relevant domain for NF2-related QoL.^25^ Further studies in a larger population of individuals with NF2 are needed to better understand pain prevalence and its causes in individuals with NF2.

We also found a high prevalence of self-reported anxiety and depression, and this is in keeping with previous studies done in different countries and healthcare systems. For example, a survey of 498 adults with NF1 showed a prevalence of depression of 55%, similar to our findings.^33^ Another survey of adults with NF1, NF2, and Schwannomatosis also found a high prevalence of depression, up to 46%.^34^ Considering the high prevalence of mental health disorders in this and other studies, routine assessment of patients with NF1 and NF2 should probably include screening for depression and related disorders. These patients may also benefit from new interventions, such as resilience training through videoconference, that have shown to be feasible and effective in improving QoL in individuals with NF1 and NF2.^35^

In our study, the main predictors of the mental component of the SF-36, which reflect mental health related QoL, were perceived physical appearance (subscore of the PedsQL NF1 module) and being divorced (compared to being single or having a partner). The influence of NF1 visibility on QoL has been mixed in previous studies. For example, the study by Wang et al.^34^ did not find a relationship between visibility (measured by an examiner) and emotional functioning in patients with NF1. However, in previous studies where participants self-rated their skin visibility, visibility was correlated with depression and QoL.^5,36^ In our study, when we looked at the Ablon index—rated by an examiner—we did not find a correlation with mental health; however, when we used self-reported physical appearance, it became a major driver of mental health related QoL. This suggests that self-perceived appearance is more relevant to patients than visibility rated by external assessors, and this is in keeping with how body image has been conceptualized.^37^ Therefore, disease visibility in NF1 should probably be measured by self-reported questionnaires, rather than by external assessors. Previous studies have found worse body image scores in women with NF1 compared to men with NF1^36^; our regression model found that women had worse scores in the PedsQL NF1 module (*P* = .026), but not in the SF-36 scores. It is possible that this reflects the lack of sensitivity of generic measures to assess body image and self-perceived appearance. Further analysis of these data by gender might provide more insight into any gender-based differences.

In the case of the NF2-specific measure, we found that hearing, balance, and outlook on life were the most affected items. This is similar to the cohort where NFTI-QoL was developed,^25^ as well as a US cohort that used this measure.^38^ There was a high correlation between the NFTI-QoL total scores and scores on generic measures such as the EQ-5D-5L and SF-36, supporting validity for the Canadian population. However, these findings are limited due to the small number of participants. Additionally, with only 8 items, the NFTI-QoL may miss other areas relevant to NF2 patients.

In addition to the small number of NF2 participants, our study was conducted in a single, tertiary academic center. Therefore, our findings may be biased toward patients who have more severe disease, who tend to be followed at speciality care centers and may not be applicable to individuals with milder disease followed in the community. Additionally, we enrolled approximately 50% of the patients screened for the study, with selection bias toward those with higher educational attainment and literacy. Unfortunately, there are no population-based data in Canada regarding individuals with NF1 and NF2, so we do not know how well our sample reflects the overall universe of Canadians living with NF1. Additionally, there are many other factors that affect QoL, such as socioeconomic status, which we did not specifically address in this study. Strengths of our study are the use of several generic and disease-specific measures that capture different aspects of QoL.

In summary, we found that adult patients with NF1 and NF2 attending a speciality clinic in Canada have reduced QoL compared to the general Canadian population. There is a high prevalence of pain and anxiety/depression in NF1 and NF2 patients. In NF1, perceived visibility is a major driver of mental well-being, while pain is a major driver of physical health. Our findings highlight the importance of multidisciplinary management of individuals with NF1 and NF2, and we suggest screening for pain and depression in routine assessments of individuals with NF. Additionally, the impact of skin manifestations in NF1 patients and its effect on mental health should be assessed individually in each patient, as self-perceived body image may differ from the skin manifestations seen by an examiner.

## Supplementary Material

vdaa003_suppl_supplementary_Figures_Tables
