# Supplementary material for: Quality of life in patients with neurofibromatosis type 1 and 2 in Canada
Source: Neurooncol Adv. 2020 Jan 10;2(Suppl 1):i141–9. doi: 10.1093/noajnl/vdaa003 (PMC7317053; doi:10.1093/noajnl/vdaa003)
Supplement: vdaa003_suppl_supplementary_Figures_Tables [file vdaa003_suppl_supplementary_figures_tables.docx]

**Quality of Life in Patients with Neurofibromatosis type 1 and 2 in Canada.**

**Supplementary Material**

**Supplementary Table 1. SF-36 Scores**

| **Domains** | **NF1**  **Mean ± Sd** | **NF2**  **Mean ± Sd** | **Canadian Norms**  **(25-34 years)**  **Mean ± Sd** |
| --- | --- | --- | --- |
| **Physical Functioning** | 77.5 ± 25.4 | 69.5 ± 29.6 | 92.4 ± 14.6 |
| **Role Physical** | 76.8 ± 27.4 | 63.8 ± 31.0 | 87.1 ± 29.3 |
| **Bodily pain** | 65.8 ± 26.4 | 62.0 ± 25.8 | 77.0 ± 21.8 |
| **General Health** | 58.1 ± 24.2 | 45.2 ± 21.9 | 79.0 ± 16.1 |
| **Energy/vitality** | 55.3 ± 21.7 | 49.4 ± 16.1 | 64.9 ± 17.7 |
| **Social Functioning** | 76.1 ± 26.2 | 58.5 ± 32.1 | 86.3 ± 20.3 |
| **Role Emotional** | 78.2 ± 25.6 | 66.2 ± 31.7 | 82.9 ± 32.3 |
| **Mental Health** | 66.8 ± 20.2 | 61.6 ± 21.4 | 75.9 ± 15.7 |
| **Physical Component Score** | 48.9 ± 10.2 | 45.5 ± 10.3 | 53.0 ± 7.2 |
| **Mental Component Score** | 47.1 ± 10.7 | 41.2 ± 12.5 | 50.1 ± 9.6 |

All domains had lower scores for NF1 and NF2 compared to Canadian normative data (t-test p<0.005 for all).

**Supplementary table 2: Mean scores in Adult Peds-QL NF1 module (n=162)**

| **DOMAINS** | **MEAN ± SD** |
| --- | --- |
| Physical function | 66 ± 36.0 |
| Emotions | 57 ±30.7 |
| Social function | 65 ±33.5 |
| Cognitive | 57 ±30.3 |
| Communications | 67 ±33.0 |
| Worry | 53 ±35.2 |
| Perceived physical appearance | 51 ± 37.1 |
| Pain | 60 ±36.4 |
| Paresthesia | 72 ±32.0 |
| Skin | 77 ±30.5 |
| Sensory | 80 ±28.8 |
| Mobility | 79 ±29.6 |
| ADLs | 92 ±20.7 |
| Fatigue | 59 ±32.8 |
| Anxiety | 80 ±29.7 |
| Sexual functioning | 85 ±29.3 |
| TOTAL Peds-QL-NF1 | 68.3 ± 17.8 |

ADLs: activities of daily living

For all dimensions, scores range from 0 to 100, with higher scores indicating better QoL

**Supplementary table 3. Regression estimates for the Peds-QL NF1 module**

|  | | | |
| --- | --- | --- | --- |
|  | **Estimate** | **SE** | **p-value** |
| **Intercept** | 128.26 | 8.28 | <0.0001* |
| **Female** | -6.54 | 2.91 | 0.027 * |
| **Age** | -0.24 | 0.10 | 0.028 * |
| **Pain Interference** | -0.93 | 0.14 | <0.0001* |
| **Known**  **Plexiform** | 0.69 | 3.03 | 0.82 |
| **Optic glioma** | -1.97 | 4.20 | 0.64 |
| **MPNST** | 8.85 | 5.25 | 0.09 |
| **Other cancers** | -6.39 | 4.53 | 0.16 |

MPNST: Malignant peripheral nerve sheath tumour. In this case history of MPNST (yes/no)

Pain interference: score on the PROMIS pain interference scale short form 8a.

*p<0.05

**Supplementary Table 4. Key variables**

| **Variable** | **Type** | **Description** |
| --- | --- | --- |
| Age | Numeric | Years |
| Sex | Categorical | Male/female |
| Employment | Categorical | Employed  Unemployed  On disability  Student  Retired  Homemaker  Other |
| Marital Status | Categorical | Single  Married or common law  Divorced/separated  Widowed  Other |
| Highest Education | Categorical | Some or completed primary  Some or completed secondary  Some or completed trade or community program  Some or completed university |
| History of MPNST | Categorical | Yes/no |
| Optic glioma | Categorical | Yes/no |
| Known Plexiform | Categorical | Yes/no  (no stratification if visible or by MRI) |
| Ablon’s Index | Ordinal | 1/2/3  Rated by clinician |
| Hearing Impairment | Ordinal | Mild/Moderate/Severe  (based on audiograms) |
| Facial Nerve Scale | Ordinal | 1/2/3/4/5/6  By examiner |
| **Patient reported outcomes** |  |  |
| SF-36 PCS | Numeric | QualityMetric algorithm  Range 4 - 71 |
| SF-36 MCS | Numeric | QualityMetric algorithm  Range 2 - 74 |
| EQ-5D-5L utility score | Numeric | Mobility  Usual activities  Self-care  Pain  Anxiety/Depression  Canadian valuation algorithm  Min possible score is -0.148 Max possible score is 0.948 |
| Peds-QL NF1 adult module | Numeric | Physical function  Emotions  Social function  Cognitive  Communications  Worry  Perceived physical appearance  Pain  Paresthesia  Skin  Sensory  Mobility  ADLs  Fatigue  Anxiety  Sexual functioning  TOTAL Peds-QL-NF1  Scores 0-100 |
| PROMIS pain interference | numeric | T-score range: 40.7 - 77 |

**Supplementary figure 1. Distribution of NFTI-QoL scores in individuals with NF2**


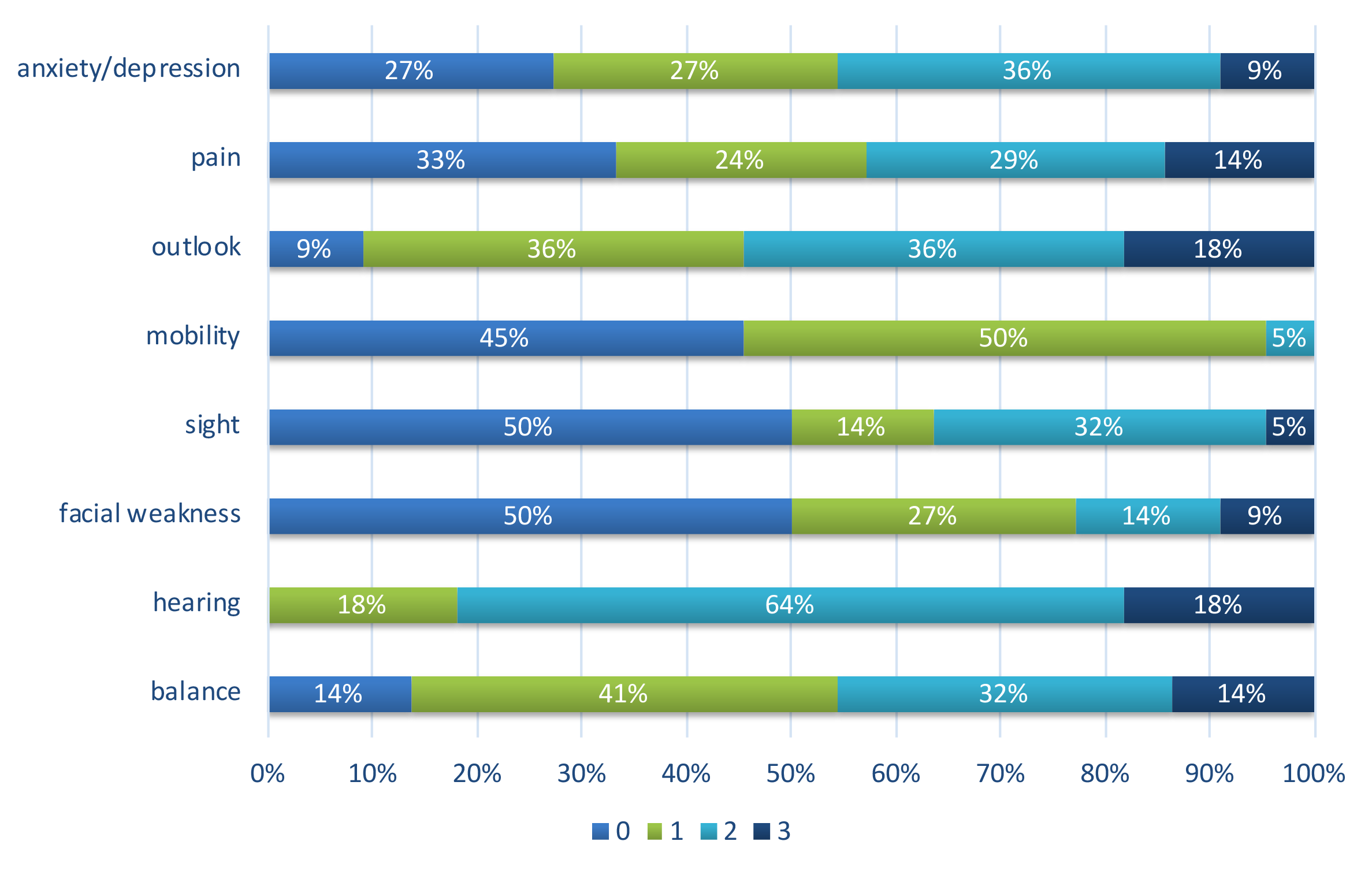


Each item is scored in a 0-3 Likert scale where higher scores indicate worse problems. In this small sample, hearing, outlook on life and balance were the most affected functions.
